# Network analysis of depressive symptoms, cognitive functioning, and life satisfaction among healthcare workers

**DOI:** 10.3389/fpsyt.2025.1586086

**Published:** 2025-07-18

**Authors:** Xiumei Hou, Yan Wang, Yang Wu, Qinge Shen, Ping Liu, Yunshuai Xu, Jicheng Dong, Yaping Wang, Min Chen, Jian Cui

**Affiliations:** ^1^ Department of Psychiatry, Shandong Daizhuang Hospital, Jining, China; ^2^ Department of Psychiatry, School of Mental Health, Jining Medical University, Jining, China; ^3^ Qingdao Mental Health Center, Qingdao University, Qingdao, Shandong, China; ^4^ The National Clinical Research Center for Mental Disorders and Beijing Key Laboratory of Mental Disorders, Capital Medical University, Beijing, China; ^5^ Beijing Anding Hospital and the Advanced Innovation Center for Human Brain Protection, Capital Medical University, Beijing, China; ^6^ Precision Medicine Laboratory, Department of Psychiatry, Shandong Daizhuang Hospital, Jining, China

**Keywords:** depression, cognitive impairment, life satisfaction, network analysis, healthcare workers

## Abstract

**Background:**

Depression and cognitive impairment among healthcare workers significantly affect their life satisfaction (LS). This study used network analysis to explore the associations between depression, cognitive symptoms, and LS in healthcare workers.

**Methods:**

A total of 655 healthcare workers were assessed using the Patient Health Questionnaire (PHQ-9), the Perceived Deficits Questionnaire-Depression (PDQ-D), and the Quality of Life Enjoyment and Satisfaction Questionnaire-Short Form (Q-LES-Q-SF). Regularized partial correlation network analysis was conducted, focusing on the strength values and predictability of each item in the network. The R software was used for statistical analysis and visualization of the network.

**Results:**

The average PHQ-9 depression score was 4.79, while the mean cognitive symptoms score was 15.38 (Our score range for all participants: PDQ-D 0 - 70; PHQ-9 0 - 27). Network analysis revealed that PDQ12 (“Trouble getting started”), PDQ13 (“Drifting”), and PDQ17 (“Remembering numbers”) were the central symptoms of the entire depression-cognition network. PHQ1 (“Anhedonia”), PHQ7 (“Concentration”), and PDQ 13 (“Drifting”) were the most critical bridge symptoms connecting depression and cognition. The three symptoms of PHQ2 (“Sad Mood”), PHQ4 (“Fatigue”), and PDQ 13 (“Drifting”) had the strongest negative correlations with LS. Gender showed no significant relationship with global network strength, edge weight distribution, or individual edge weights.

**Conclusion:**

This network analysis identified several central symptoms, including “Trouble getting started”, “Drifting”, and “Remembering numbers”. It also identified bridge symptoms such as “Anhedonia”, “Concentration”, and “Drifting”. These findings provide important evidence for the development of targeted interventions. Furthermore, measures such as improving emotional management, increasing rest periods, and providing psychological support may help alleviate fatigue and low mood, enhance attentional functioning, and ultimately improve life satisfaction among healthcare workers.

## 1 Introduction

Healthcare workers represent the backbone of global health systems, yet they are subjected to numerous challenges, including heavy workloads, irregular schedules, and occupational risks (1, 2). Long - term exposure to these stressful environments has a significant effect on the physical, mental and social health of medical personnel (3). Psychological symptoms include depression and anxiety (4). Studies estimate that approximately 19.01% of physicians experience both anxiety and depressive symptoms, 25.67% report anxiety symptoms, and 28.13% present with depressive symptoms (5). Given that mental health issues among healthcare workers have become a significant threat, this problem has been attracting increasing attention worldwide as a critical public health challenge (6). Consequently, it is particularly important to conduct in-depth investigations into the factors that influence the mental health of healthcare workers.

Depressive symptoms can impose substantial distress and dysfunction in individuals. Beyond emotional difficulties, neurocognitive dysfunction emerges as an independent contributor to diminished psychosocial functioning (7). Symptoms of cognitive impairment were also observed in patients with a first episode of depression, including poor memory, psychomotor speed, attention, visual learning, and executive function (7). Over the past two decades, there has been a great deal of focus on identifying and overcoming cognitive biases in physician diagnostic errors (8). Errors in clinical judgment and decision-making resulting from systematic errors in thought processes can have dire consequences for healthcare, and cognitive bias among healthcare providers has been identified as a significant contributor to healthcare adverse events (9). Life satisfaction refers to the degree to which an individual enjoys life (10). There are many factors affecting life satisfaction, including social factors, psychological factors, personality factors and so on (11). Bora et al. reviewed over 20 studies and concluded that cognitive dysfunction (12)—including aspects such as global cognition, attention, memory, and executive function—correlates with psychosocial impairment, which affects social and occupational functioning, overall global functioning, and life satisfaction (LS) (13). Cognitive impairment not only restricts the daily life and work ability of healthcare workers, but also affects their emotion regulation and interpersonal communication, thus further reducing their life satisfaction. Due to long-term exposure to a high-pressure work environment (such as taking care of critically ill patients, heavy workloads, high risk of infection, ethical dilemmas, etc.), burnout occurs. Burnout is manifested in both the physical and emotional aspects. On the one hand, medical staff feel physically and mentally exhausted. This reduces their professional effectiveness and personal sense of accomplishment, and affects their overall quality of life (14, 15). Optimism, self-regulation ability, positive emotions, and life satisfaction as a health asset are all core elements of positive psychology. These factors can jointly promote physical and mental health and strengthen positive self-awareness (16). From the perspective of network analysis, these related factors can be conceptualized as interconnected nodes in complex systems.

Network analysis is a method that can be used to quantify different symptoms and their relationships in a complex system (17). In network analysis, different variables are represented by nodes, and the relationship between variables is the connected edge between nodes (18). Node centrality statistics (such as strength, expected impact (EI)) are used to measure the characteristics of nodes and to identify central (influential) symptoms in the network. Central symptoms refer to symptoms that are at the core of the symptom network structure and have a high degree of connectivity. It is suggested that it has a strong connection with other symptoms, either directly or indirectly. By identifying core symptoms and developing precise intervention strategies, it is possible to exert a broad impact on the entire symptom network, thereby more effectively improving overall symptom presentation (19). Bridge symptoms increase the risk of transmission from one disease to another. Bridge symptoms can provide key entry points for intervention and treatment. Network analysis suggested that the central symptoms and bridge symptoms connected the symptoms of comorbidities, and suggested the key clues of comorbidities, so as to provide more targeted intervention and personalized comprehensive treatment for the disease (20, 21).

Previous studies have applied network analysis to explore the association between cognitive performance and depressive symptoms in older adults from the general population. Findings revealed that “feeling blue/depressed”, “everything was an effort”, and “attention and calculation” emerged as core symptoms in the depression-cognition network. Critical bridging symptoms connecting depression and cognitive domains included “naming”, “difficulty concentrating”, and “language”. Moreover, “sleep disturbances” demonstrated the strongest direct link to quality of life (22). Similarly, symptoms such as “fatigue”, “trouble relaxing”, and “nervousness” were found to have the most pronounced negative associations with quality of life (23). Although network analysis has been extensively applied to various subgroups, its application to healthcare workers remains limited. Specifically, the relationship between individual depressive symptoms, cognitive impairments, and LS in this population has not yet been thoroughly investigated.

This study aimed to identify central and bridge symptoms within a depression–cognition network and examine their associations with life satisfaction among healthcare workers. By identifying central and bridging symptoms, this research seeks to provide evidence for the development of targeted interventions to improve healthcare workers’ mental health and LS.

## 2 Method

### 2.1 Participants and procedure

From September 13, 2022 to October 25, 2022, an online cross-sectional study was conducted in Shandong Daizhuang Hospital and Qingdao Mental Health Center to investigate the mental health status of healthcare workers. Data were collected using an online questionnaire hosted on the professional survey platform Wenjuanxing (www.wjx.cn) and distributed via the WeChat social media platform. Participants were required to meet the following inclusion criteria: 1. Healthcare workers capable of understanding the assessment; 2. Aged 18 years or older. Exclusion criteria included: 1. previously diagnosed with bipolar affective disorder, schizophrenia, schizoaffective disorder, or other comorbid psychiatric disorders. This study involved the recruitment of 655 healthcare workers to participate and the successful completion of questionnaire collection. Our sample size (N = 655) was determined based on precedents from comparable studies analyzing networks of similar complexity (24–27). All participants provided electronic informed consent, and the study received approval from the Ethics Committee of Shandong Daizhuang Hospital (Ethics number: 202208KS-1). All participants provided electronic informed consent, and the study received approval from the Ethics Committee of Shandong Daizhuang Hospital (Ethics number: 202208KS-1). The data presented in this study were collected during the same period as those in our previous work, but were analyzed with a different focus and methodology to explore new research questions (28).

### 2.2 Measurements

The Perceived Deficit Questionnaire for Depression (PDQ-D) assesses cognitive function through a combination of subjective and objective measures (29). It consists of 20 items, with a total score ranging from 0 to 80. Higher scores indicate greater perceived cognitive impairment. The Patient Health Questionnaire (PHQ-9) is used to assess depressive symptoms and consists of 9 items (30). The total score ranges from 0 to 27, with a higher score indicating more severe depressive symptoms. Additionally, the Life Satisfaction Enjoyment and Satisfaction Questionnaire-Short Form (Q-LES-Q-SF) evaluates the enjoyment and satisfaction of daily life (31). It comprises 16 items, with the total score derived from the first 14 items; a higher score reflects greater enjoyment and satisfaction with life. The final two items, which address medication use and overall life satisfaction, are not included in the total score. These scales have been fully validated in the Chinese population and possess good validity and reliability (32, 33).

### 2.3 Statistical analysis

#### 2.3.1 Network analysis

R software was used for network analysis, including network estimation, centrality index calculation, and network accuracy and stability assessment (version 4.3.0) (34). In the network analysis of depression and cognitive symptoms, nodes represent the severity levels of depressive and cognitive symptoms, while the edges between two nodes indicate the associations between the symptoms. Edge thickness reflects the strength of associations, with red edges representing negative correlations and green edges indicating positive correlations. To estimate the network model, the Sparse Graph Gaussian Model (GGM) was employed in conjunction with the Least Absolute Shrinkage and Selection Operator (LASSO) method. LASSO was utilized to regularize the network model (35), thereby reducing the number of pseudo-edges and enhancing the interpretability of the results. The Extended Bayesian Information Criterion (EBIC) was applied to select the optimal model, with the EBIC hyperparameter set at 0.5, as recommended in previous studies (36).

#### 2.3.2 Central symptoms

The centrality index of strength was used to evaluate the influence of each node within the network. Nodes with higher strength values were identified as having a more prominent influence on the overall network structure. For this analysis, we used the bootnet (35) and qgraph (18) in R package. To identify bridge symptoms that serve as key connectors between two symptom communities, the bridge strength centrality index was employed. This approach highlights symptoms playing critical roles in linking distinct symptom clusters. We estimate the predictability index for each node, which reflects the likelihood that the state of a given node can be inferred from the states of its neighboring nodes. The package “mgm” was used to estimate the predictability of each node (37). Furthermore, to explore depressive and cognitive symptoms associated with LS, we used the “flow” function from the R package qgraph (18, 38).

#### 2.3.3 Stability and accuracy of the network

To assess the stability and accuracy of the network, we utilized the bootnet package. To evaluate edge accuracy, 95% confidence intervals for edge weights were generated through bootstrapping. The narrower the interval, the higher the accuracy (39). Next, to assess the stability of centrality indices, we calculated the correlation stability coefficient (CS-C) using the case-dropping bootstrap method. CS-C reflects the maximum proportion of samples that can be removed. In this case, each centrality index in both networks can show a correlation of 0.7 or higher with 95% probability. Previous studies suggest that the correlation coefficient should exceed 0.5 and not fall below 0.25 (35). Additionally, we conducted a difference test on strength centrality and edge weights between nodes (a significance level of α=0.05), with *P* < 0.05 considered statistically significant. In the current study, 1,000 bootstrap iterations were used in all cases.

#### 2.3.4 Gender network comparison

We investigated whether network characteristics differed between male and female health care worker participants using the Network Comparison Test (NCT) package, which is based on permutation tests involving 1000 pairs of subsamples (male vs. female). This analysis assessed the overall network structure, global strength, and differences at each edge between subsamples. After adjusting for multiple comparisons using the Holm-Bonferroni correction, we assessed the strength difference of each edge in the male and female networks (40). All tests were performed using the R package ‘NetworkComparisonTest’ version 2.2.1.

## 3 Result

### 3.1 Characteristics of study samples

This study included a total of 680 participants, of whom 655 met the inclusion criteria and successfully completed the assessment. Among these participants, 459 (70.08%) were women, 647 (98.87%) were with college education or above, and 496 (75.73%) were Married or cohabiting (**Supplementary Table 1**). Descriptive statistics, including means, standard deviations, skewness, and kurtosis for all PHQ-9 and PDQ-D item scores, are provided in **Table 1**.

**Table 1:** Edge weights and predictabilities of PHQ-9 and PDQ-D items (N=655).

Item ID	Item Content	M	SD	Skew	Kurtosis	Strength	Pred
PHQ1	Anhedonia	0.65	0.72	1.10	1.28	0.91	0.60
PHQ2	Sad Mood	0.56	0.68	1.21	1.70	0.87	0.59
PHQ3	Sleep problems	0.75	0.81	1.04	0.78	0.66	0.73
PHQ4	Fatigue	0.85	0.74	0.71	0.46	0.93	0.63
PHQ5	Appetite	0.65	0.77	1.14	0.99	0.67	0.76
PHQ6	Guilt	0.40	0.64	1.73	3.26	0.82	0.64
PHQ7	Concentration	0.47	0.69	1.50	2.07	0.94	0.60
PHQ8	Motor problems	0.32	0.61	2.11	4.64	0.91	0.62
PHQ9	Suicide ideation	0.14	0.47	3.88	16.52	0.53	0.71
PDQ1	Lose train of thought	1.01	0.81	0.73	0.77	0.79	0.62
PDQ2	Difficulty remember name	0.97	0.89	0.77	0.18	0.71	0.68
PDQ3	Forget what	0.92	0.80	0.65	0.11	0.88	0.57
PDQ4	Trouble things organized	0.74	0.80	1.07	1.21	0.90	0.59
PDQ5	Talk focus difficulty	0.70	0.75	0.88	0.58	0.92	0.53
PDQ6	Forget already done	0.86	0.77	0.61	0.06	0.94	0.54
PDQ7	Forget appointments	0.54	0.71	1.26	1.51	0.80	0.60
PDQ8	Difficulty planning	0.69	0.81	1.18	1.33	0.93	0.55
PDQ9	Reading concentration difficulty	0.84	0.84	0.83	0.37	0.87	0.56
PDQ10	Forget things	0.64	0.75	1.11	1.17	0.91	0.53
PDQ11	Forget date	0.91	0.89	0.95	0.73	0.86	0.62
PDQ12	Trouble getting started	0.72	0.83	1.30	2.00	0.97	0.52
PDQ13	Drifting	0.91	0.84	0.93	1.21	1.00	0.51
PDQ14	Forget talked about	0.60	0.72	1.05	0.75	0.86	0.57
PDQ15	Forget routine things	0.68	0.77	1.18	1.73	0.90	0.57
PDQ16	Mind totally blank	0.65	0.80	1.32	1.94	0.88	0.54
PDQ17	Remembering numbers	0.76	0.82	0.95	0.62	0.96	0.53
PDQ18	Forget things	0.83	0.86	0.98	0.77	0.89	0.55
PDQ19	Forget medication	0.63	0.79	1.27	1.59	0.75	0.65
PDQ20	Trouble making decisions	0.79	0.87	1.24	1.69	0.92	0.57

M, Mean; SD, Standard deviation; Skew, Skewness; PHQ-9, Patient Health Questionnaire; PDQ-D, Perceived Deficit Questionnaire for Depression; Pred, Predictability.

### 3.2 Network structure

The mean PDQ-D and PHQ-9 total scores in the participants were 15.38 and 4.79 (Our score range for all participants: PDQ-D 0 - 70; PHQ-9 0 - 27). Tests of item informativeness indicated no item ratings were < 2.5 SD from the average amount of information (i.e., SD) of PHQ-9 (M _SD_ = 0.68 ± 0.10) or PDQ-D (M _SD_ = 0.81± 0.05). The redundancy analysis of the items revealed that none of the PHQ-9 or PDQ-D items were found to overlap significantly or be redundant with other items in the measurement. Therefore, all PHQ-9 or PDQ-D items were retained in the analyses.

**Figure 1:**
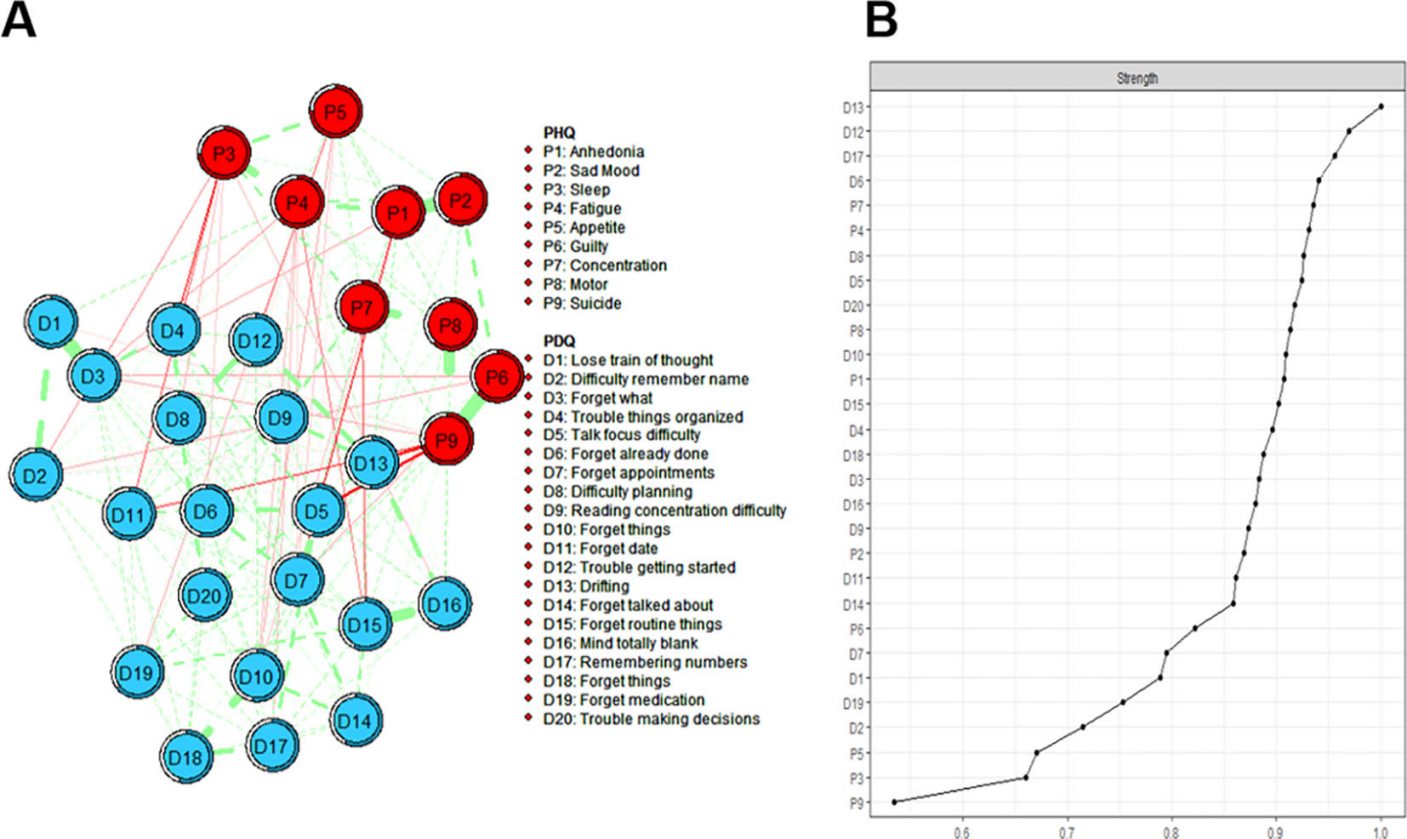
Estimated network structure of depressive and cognitive symptoms and the corresponding centrality of each node. **(A)** The depressive and cognitive impairment network structure. The different-size circles represent different strength of the nodes, while the width and saturation of edges indicate the connections and directions (i.e., green: positive correlation; red: negative correlation). The ring around each node indicates the predictability (a fully filled dark ring would indicate that 100% of the symptom’s variance is explained by its intercorrelations with the other symptoms in the network). PDQ-D=Perceived Deficit Questionnaire for Depression, PHQ-9 = Patient Health Questionnaire. **(B)** The strength of symptoms in the depressive and cognitive impairment network.

**Figure 2:**
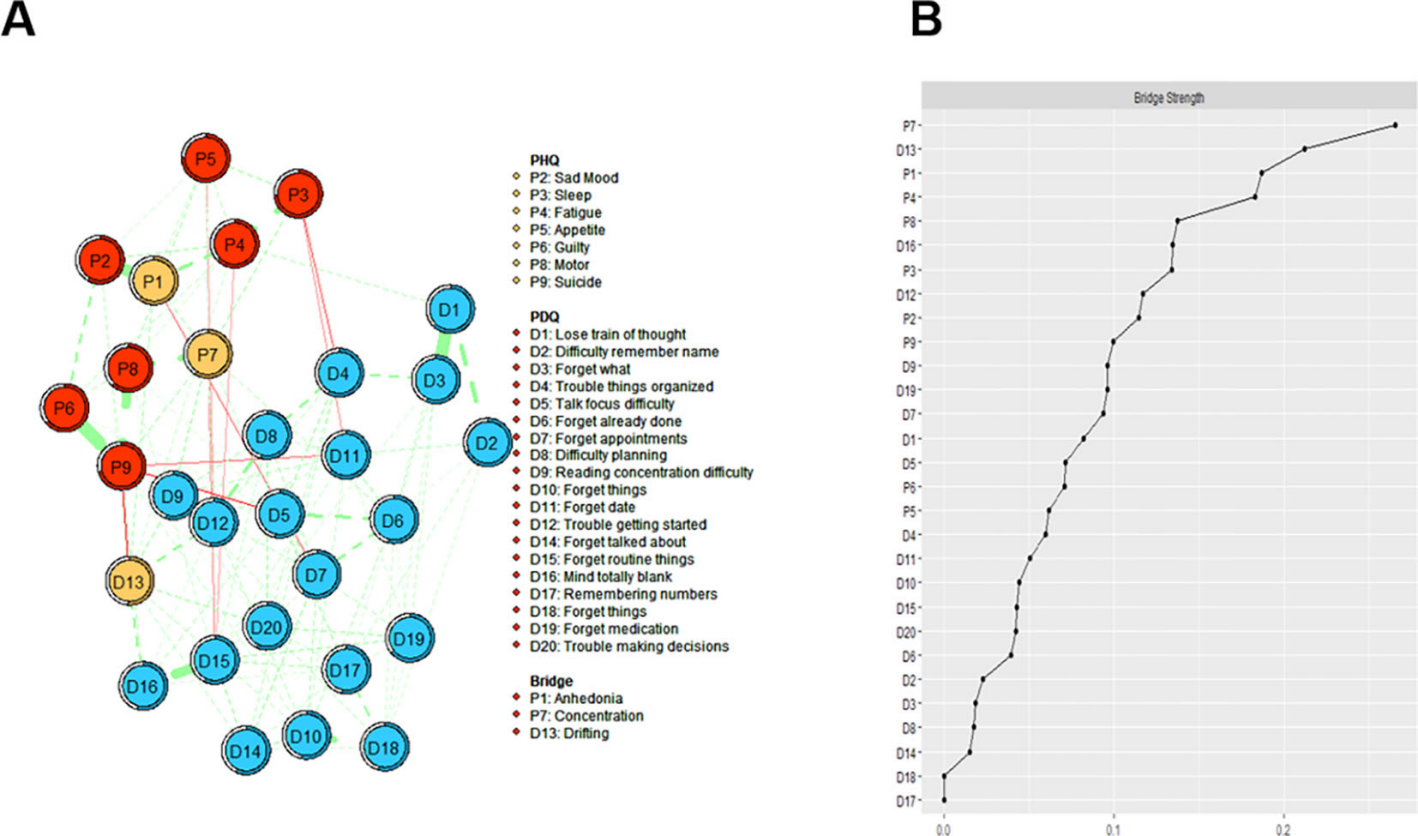
Estimated network structure of comorbid depressive and cognitive symptoms and the corresponding bridge symptoms. **(A)** The depressive and cognitive impairment network structure shows the bridge symptoms. The different-size circles represent different strength of the nodes, while the width and saturation of edges indicate the connections and directions (i.e., green: positive correlation; red: negative correlation). The ring around each node indicates the predictability (a fully filled dark ring would indicate that 100% of the symptom’s variance is explained by its intercorrelations with the other symptoms in the network). **(B)** The bridge strength of symptoms in the depressive and cognitive impairment network. PDQ-D=Perceived Deficit Questionnaire for Depression, PHQ-9 = Patient Health Questionnaire.

In the depression and cognitive symptom network structure for healthcare workers is shown in **Figure 1**. In the cognitive symptom and depression network, PDQ12 (“Trouble getting started”), PDQ13 (“Drifting”), and PDQ17 (“Remembering numbers”) demonstrate the highest strength centrality, indicating that these symptoms are the most influential within the model. Additionally, PHQ1 (“Anhedonia”), PHQ7 (“Concentration”), and PDQ13 (“Drifting”) had the most important bridge strength, linking cognitive deficits and depressive symptoms in **Figure 2** and detailed in **Supplementary Table 2**.

**Figure 3:**
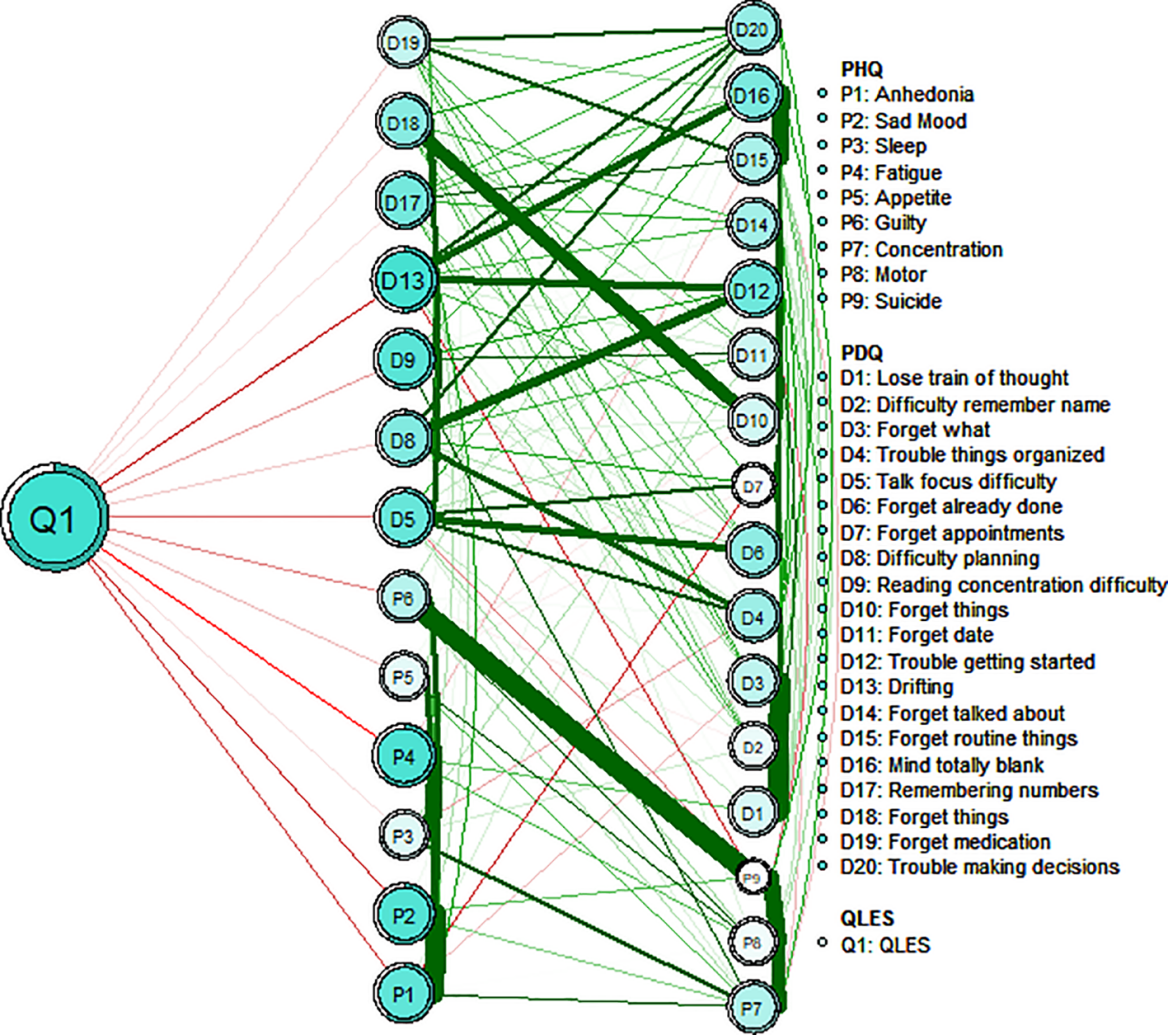
Flow network of life satisfaction, depressive symptoms and cognitive performance.

**Figure 3** depicts the flow diagram presenting how “QLES” is connected to cognitive deficits and depressive symptoms in the network model. The 15 nodes located in the middle of the figure are directly related to QLES, while the remainder 14 nodes are indirectly related to QLES. The symptoms showing the strongest direct associations with QLES were PHQ2 (“Sad Mood”), PHQ4 (“Fatigue”), and PDQ 13 (“Drifting”). We have performed additional sub - network analyses. This network focuses on the independent associations between depressive symptoms (measured by PHQ - 9) and life satisfaction (Q - LES - Q - SF) (**Supplementary Figures 1, 2**).

### 3.3 Accuracy and stability of the network

**Figure 4:**
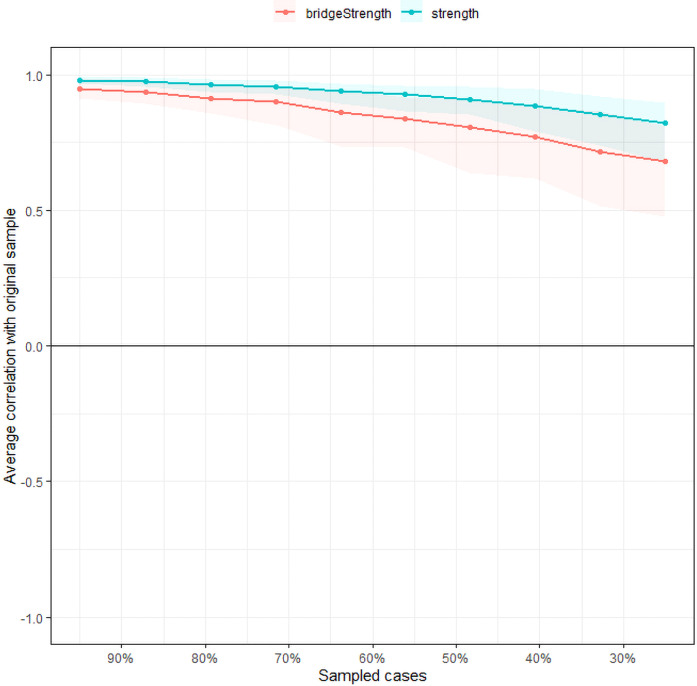
Stability of the network structure of comorbid depressive and cognitive symptoms estimated by a case-dropping bootstrapped method. The x-axis represents the percentage of cases of the original sample used at each step. The y-axis represents the average of correlations between the centrality indices in the original network and the centrality indices from the re-estimated networks after excluding increasing percentages of cases. The line indicates the correlations of strength and bridge strength.

Regarding network stability, the case-dropping bootstrap procedure showed that the rank order of node strength centrality remained stable after dropping different proportions of the sample as shown in **Figure 4** (i.e., CS-coefficient = 0.672). This indicates that when dropping up to 67.2% of the sample, the order of the symptoms in strength was still correlated with the original one (*r* = 0.7). The bootstrap difference test revealed that the majority of edge weight comparisons are statistically significant. The accuracy of edge weights calculated by bootstrap method shows that the bootstrapped 95% confidence interval of edge weights were narrow, while most of the edge values were non-zero, indicating that the network structure was accurate enough (**Supplementary Figure 3**).

### 3.4 Gender network comparison

Comparison of network models between female (n = 459) and male (n = 196) healthcare workers showed no statistically significant differences in global network strength (network strength: 13.87 for females and 13.83 for males, S = 0.032, *p* = 0.887) and distribution of edge weights (M = 0.21, *p* = 0.545; **Supplementary Figures 4, 5**).

## 4 Discussion

This study explored the network structural relationship between depression and cognitive symptoms comorbidity in healthcare workers. Analyses indicated nodes “Trouble getting started”, “Drifting” and “Remembering numbers” were central symptoms in the network. These symptoms are crucial for understanding the network’s overall structure in this population. Additionally, “Anhedonia”, “Concentration”, and “Drifting” had the most important bridge strength, linking depressive symptoms and cognitive deficits. Furthermore, “Sad Mood”, “Fatigue”, and “Drifting” were directly associated with lower LS among healthcare workers.

“Drifting” was associated with emotional and cognitive symptoms among healthcare workers. “Drifting” was identified as both a central and a bridge symptom in this network analysis, as indicated by its high strength centrality and bridge centrality values. Furthermore, flow network analysis revealed a robust association between “Drifting” and LS. This symptom is characterized by absent-mindedness and difficulty concentrating, which are indicative of impaired attention and cognitive functioning in healthcare workers. Higher-order cognitive functions such as executive functioning and attentional ability are closely linked to both physical and mental well-being (41). Attention is essential for selectively focusing on external stimuli (42). The study has also reported similar findings, suggesting that attentional deficits occupy a central position in the network and have strong associations with other cognitive domains (such as memory, executive function) and affective symptoms (such as depressive mood) (22). Exteroceptive attention demonstrates a positive correlation with self-awareness and subjective well-being, enabling individuals to disengage from aversive stimuli for emotional regulation. Enhanced attentional capacity may further facilitate emotional downregulation, delay gratification, reduce impulsivity, and contribute to more judicious decision-making processes. Mediation analysis showed that attention mediated the relationship between agitation (e.g., anxiety or depression) and Quality of Life (QoL) (43). According to Easterbrook’s theory, emotional arousal can lead to narrow attention (44), limiting individuals’ ability to manage work, learning, social interactions, and emotion regulation. With the aggravation of attention problems, the individual’s daily functioning is limited, which in turn reduces the overall LS (45). Emotional awareness is a key part of health and emotion regulation, and internal feedback (including physical signals and emotional awareness) can guide people to the best strategy. Higher frequency of switching strategies predicted higher life satisfaction (46). These findings highlight the importance of enhancing attention in improving healthcare workers’ mood and cognitive performance to enhance life satisfaction.

“Trouble getting started” and “Remembering numbers” were also prominent central symptoms in the depression-cognition network. Individuals with depression often struggle with initiating tasks, a difficulty closely associated with motivational deficits and low mood (47). Cognitive control deficits in depression can be viewed as changes in the decision-making processes underlying the allocation of cognitive control, caused by alterations in key components of motivation (48). “Remembering numbers” highlights working memory deficits, especially number memory. Previous studies have shown that memory is a complex cognitive function that requires a trade-off between stability and flexibility. The crucial sub-processes of working memory include the ability to maintain stored information in the absence of irrelevant distractions and the ability to update limited storage capacity in response to new information (49). Memory impairments in depression typically manifest as poor attention and short-term memory, leading to challenges in daily activities (50, 51). Related studies have further verified that cognitive impairment, especially in working memory and attention, often leads to difficulties in performing daily tasks (52). Depression and cognitive deficits are prevalent among healthcare workers and are considered core features of the disorder (53). These findings further validate our findings regarding “Trouble getting started” and “Remembering numbers” as a central symptom in our network model of depressive symptoms and cognitive performance in healthcare workers. It has been shown in network theory that targeting the central symptom may reduce the severity of other symptoms in the model (54). Interventions focusing on improving working memory and executive function may, therefore, reduce the burden of depression and cognitive symptoms among healthcare workers, ultimately enhancing their quality of life.

In addition, “Anhedonia” and “Concentration” were found to be the key bridge symptoms connecting depressive and cognitive symptoms in the network. Anhedonia as a major core symptom of major depressive disorder, conforming to the Diagnostic and Statistical Manual of Mental Disorders 5 (DSM-5) diagnosis (47). “Anhedonia” is closely related to the dysfunction of dopaminergic system, which not only affects the emotional state of patients, but also may further aggravate the impairment of social function through the interaction with cognitive function (55). At the same time, concentration is a closely related cognitive structure, and the state of attention is conducive to the deep processing of information (56). Reduced cognitive function may make patients face more difficulties in performing daily tasks, and may also affect their ability to regulate emotions, thereby aggravating depressive symptoms (57). Previous studies have emphasized the long-term interplay between depressive symptoms and cognitive dysfunction. Meta-analyses have found that individuals with depressive symptoms exhibit moderate cognitive impairments, particularly affecting executive function, memory, and attention (58). Cai et al. explained that anhedonia and concentration as bridge symptoms, the bridge symptoms can trigger and maintain the comorbid psychiatric syndromes (59). Therefore, addressing bridge symptoms could be critical for alleviating these interconnected syndromes, enabling more targeted interventions.

In the network model of depressive symptoms and cognitive performance, we also found that “Sad Mood” and “Fatigue” symptoms were negatively correlated with LS. Sadness is usually one of the important manifestations of depression, and fatigue is a common physiological symptom among healthcare workers (14, 60). Labor is affected by working conditions and leads to the aggravation of their emotions, which has a negative impact on the health of medical staff (61). The working environment of healthcare workers is usually stressful. Long hours of high-intensity work and the emotional stress of patients’ conditions can easily lead to psychological and physical fatigue (62, 63). According to relevant studies, Healthcare workers often suffer from fatigue caused by overwork, which not only affects their physical health, but also causes emotional problems such as sadness and anxiety (6, 64). Sad emotions are often associated with negative affect and low self-esteem of individuals, which may lead to a lack of positive outlook on life among healthcare workers (65). These symptoms may make healthcare workers feel more powerless in the face of work stress and daily life, thus affecting their overall evaluation of life (66, 67). To a certain degree, our research results corroborate these findings. Therefore, improving the emotional state of healthcare workers and reducing work stress may be important factors for improving LS and cognitive ability of healthcare workers. In addition, when comparing our research findings with existing literature, we have noticed that many studies have reported gender differences in the manifestation of depressive and cognitive symptoms (68). From a biological perspective, fluctuations in female sex hormones and the dynamics of related neurotransmitters make women more susceptible to depression (69). Psychologically speaking, different genders may also employ distinct psychological defense mechanisms, which may affect the presentation of symptoms (70). However, our network analysis shows that there are no significant differences between genders. This result indicates that, in our study, the underlying mechanisms influencing the relationships among depressive symptoms, cognitive performance, and other variables may operate in a similar manner across different genders. At the same time, it confirms that the associations and patterns revealed by our network analysis are relatively stable regardless of gender.

This study utilized validated standardized tools and network analysis methods to visualize the potential connections between cognitive and depressive symptoms in healthcare workers. Despite the advantage of having a representative sample population, there are several potential limitations. First, although gender did not have a significant effect on the primary outcome, the majority of participants in this study were women. Moreover, the network structure in this study was specific to healthcare workers in Shandong, China. Therefore, the further generalization of the study results is limited. Second, as a cross-sectional study, it is unable to explore the dynamic changes or causal relationships between individual symptoms, highlighting the need for future longitudinal research. In the future, we can explore the dynamic effects of adjusting core symptoms and bridge symptoms on life satisfaction through long-term follow-up and intervention experiments, so as to provide more solid evidence for further understanding and improving the mental health of medical staff. Finally, the questionnaire assessments in this study were primarily based on self-report, which may be affected by recall or reporting biases. Future research should consider incorporating objective cognitive tests. Although participants who reported having a psychiatric diagnosis or taking medications were excluded, the absence of structured clinical interviews (such as the Mini International Neuropsychiatric Interview, MINI) means that undiagnosed cases may still have been included in the study.

## 5 Conclusions

In summary, the findings of this network study highlight the associations between depressive symptoms and cognitive performance in healthcare workers. The central symptoms identified by the model include “Trouble getting started”, “Drifting”, and “Remembering numbers”. Interventions targeting bridge symptoms such as “Anhedonia”, “Impaired concentration”, and “Drifting” could potentially alleviate depressive and cognitive symptoms in healthcare workers. Moreover, some measures were taken to improve the attention, emotion regulation and sleep of medical staff, so as to improve their life satisfaction. For example, in the aspect of attention regulation, an example of computer-based attention training program was added. In the level of emotion management, the application ideas of mindfulness-based cognitive therapy (MBCT) and emotional release therapy (EFT) were introduced. At the same time, based on the work characteristics of doctors, specific measures such as optimizing the shift system and extending the rest time during work intervals can be adopted to rationally plan the rest arrangements in the workplace.

## Data Availability

The raw data supporting the conclusions of this article will be made available by the authors, without undue reservation.
