# Supplementary material for: Network analysis of depressive symptoms, cognitive functioning, and life satisfaction among healthcare workers
Source: Front Psychiatry. 2025 Jul 18;16:1586086. doi: 10.3389/fpsyt.2025.1586086 (PMC12315560; doi:10.3389/fpsyt.2025.1586086)
Supplement: Supplementary file 1 [file Table1.docx]

**Supplementary materials**

Table S1. Socio-demographic features of participants (N = 655)

Table S2. Correlation matrix of the PHQ-9 and PDQ-D items

Figure S1. Estimated network model for the association between depressive symptoms and life satisfaction.

Figure S2. Estimated network model for the association between cognitive performance and life satisfaction.

Figure S3. Nonparametric bootstrapped accuracy test for edge-weights of PHQ-9 and PDQ-D items

Figure S4. Estimated network models for depressive and cognitive impairment symptoms in male and female participants

Figure S5. Statistical difference map of global strength and maximum of difference

**Table S1.** Socio-demographic features of participants (N = 655)

| Variables | N (%) |
| --- | --- |
| Age (31 - 40 years old) | 257 (39.23) |
| Female gender | 459（70.08） |
| Education level |  |
| Associate Degree | 105 (16.03) |
| Undergraduate Degree | 378 (57.71) |
| Master's Degree or above | 164 (25.04) |
| Married/cohabiting | 496 (75.73) |
| Low income (monthly income < 5,000RMB) | 216 (32.98) |

**Table S2.** Correlation matrix of the PHQ-9 and PDQ-D items

|  | PHQ-1 | PHQ-2 | PHQ-3 | PHQ-4 | PHQ-5 | PHQ-6 | PHQ-7 | PHQ-8 | PHQ-9 | PDQ-1 | PDQ-2 | PDQ-3 | PDQ-4 | PDQ-5 | PDQ-6 | PDQ-7 | PDQ-8 | PDQ-9 | PDQ-10 | PDQ-11 | PDQ-12 | PDQ-13 | PDQ-14 | PDQ-15 | PDQ-16 | PDQ-17 | PDQ-18 | PDQ-19 | PDQ-20 |
| --- | --- | --- | --- | --- | --- | --- | --- | --- | --- | --- | --- | --- | --- | --- | --- | --- | --- | --- | --- | --- | --- | --- | --- | --- | --- | --- | --- | --- | --- |
| PHQ-1 | 0.000 |  |  |  |  |  |  |  |  |  |  |  |  |  |  |  |  |  |  |  |  |  |  |  |  |  |  |  |  |
| PHQ-2 | 0.349 | 0.000 |  |  |  |  |  |  |  |  |  |  |  |  |  |  |  |  |  |  |  |  |  |  |  |  |  |  |  |
| PHQ-3 | 0.027 | 0.064 | 0.000 |  |  |  |  |  |  |  |  |  |  |  |  |  |  |  |  |  |  |  |  |  |  |  |  |  |  |
| PHQ-4 | 0.252 | 0.116 | 0.265 | 0.000 |  |  |  |  |  |  |  |  |  |  |  |  |  |  |  |  |  |  |  |  |  |  |  |  |  |
| PHQ-5 | 0.099 | 0.075 | 0.161 | 0.116 | 0.000 |  |  |  |  |  |  |  |  |  |  |  |  |  |  |  |  |  |  |  |  |  |  |  |  |
| PHQ-6 | 0.000 | 0.190 | 0.000 | 0.088 | 0.078 | 0.000 |  |  |  |  |  |  |  |  |  |  |  |  |  |  |  |  |  |  |  |  |  |  |  |
| PHQ-7 | 0.086 | 0.054 | 0.088 | 0.007 | 0.114 | 0.126 | 0.000 |  |  |  |  |  |  |  |  |  |  |  |  |  |  |  |  |  |  |  |  |  |  |
| PHQ-8 | 0.032 | 0.000 | 0.012 | 0.031 | 0.058 | 0.212 | 0.283 | 0.000 |  |  |  |  |  |  |  |  |  |  |  |  |  |  |  |  |  |  |  |  |  |
| PHQ-9 | 0.000 | 0.026 | 0.000 | 0.000 | 0.000 | 0.170 | 0.040 | 0.272 | 0.000 |  |  |  |  |  |  |  |  |  |  |  |  |  |  |  |  |  |  |  |  |
| PDQ-1 | 0.000 | 0.000 | 0.000 | 0.074 | 0.008 | 0.000 | 0.000 | 0.000 | 0.000 | 0.000 |  |  |  |  |  |  |  |  |  |  |  |  |  |  |  |  |  |  |  |
| PDQ-2 | 0.000 | 0.000 | 0.023 | 0.000 | 0.000 | 0.000 | 0.000 | 0.000 | 0.000 | 0.210 | 0.000 |  |  |  |  |  |  |  |  |  |  |  |  |  |  |  |  |  |  |
| PDQ-3 | -0.018 | 0.000 | 0.000 | 0.000 | 0.000 | 0.000 | 0.000 | 0.000 | 0.000 | 0.305 | 0.064 | 0.000 |  |  |  |  |  |  |  |  |  |  |  |  |  |  |  |  |  |
| PDQ-4 | 0.000 | 0.000 | -0.031 | 0.014 | 0.000 | 0.015 | 0.000 | 0.000 | 0.000 | 0.017 | 0.078 | 0.147 | 0.000 |  |  |  |  |  |  |  |  |  |  |  |  |  |  |  |  |
| PDQ-5 | 0.000 | 0.016 | 0.000 | 0.000 | 0.002 | 0.000 | 0.046 | 0.000 | -0.006 | 0.034 | 0.000 | 0.000 | 0.101 | 0.000 |  |  |  |  |  |  |  |  |  |  |  |  |  |  |  |
| PDQ-6 | 0.000 | 0.009 | 0.008 | 0.000 | 0.000 | 0.000 | -0.021 | 0.000 | 0.000 | 0.120 | 0.029 | 0.071 | 0.048 | 0.151 | 0.000 |  |  |  |  |  |  |  |  |  |  |  |  |  |  |
| PDQ-7 | -0.047 | 0.000 | 0.000 | -0.007 | 0.000 | 0.000 | 0.000 | 0.013 | 0.027 | 0.000 | 0.030 | 0.000 | 0.000 | 0.144 | 0.079 | 0.000 |  |  |  |  |  |  |  |  |  |  |  |  |  |
| PDQ-8 | 0.000 | 0.000 | 0.000 | 0.000 | 0.000 | 0.000 | 0.017 | 0.000 | 0.000 | 0.000 | 0.016 | 0.000 | 0.199 | 0.081 | 0.034 | 0.148 | 0.000 |  |  |  |  |  |  |  |  |  |  |  |  |
| PDQ-9 | 0.006 | 0.000 | 0.000 | 0.000 | 0.000 | 0.000 | 0.090 | 0.000 | 0.000 | 0.000 | 0.000 | 0.000 | 0.031 | 0.068 | 0.039 | 0.000 | 0.135 | 0.000 |  |  |  |  |  |  |  |  |  |  |  |
| PDQ-10 | 0.000 | 0.000 | 0.000 | 0.000 | 0.000 | 0.024 | 0.000 | 0.020 | 0.000 | 0.000 | 0.000 | 0.000 | 0.000 | 0.062 | 0.098 | 0.076 | 0.052 | 0.000 | 0.000 |  |  |  |  |  |  |  |  |  |  |
| PDQ-11 | 0.000 | 0.000 | 0.040 | 0.000 | 0.000 | 0.000 | 0.000 | 0.000 | -0.010 | 0.023 | 0.134 | 0.059 | 0.000 | 0.000 | 0.143 | 0.007 | 0.042 | 0.107 | 0.089 | 0.000 |  |  |  |  |  |  |  |  |  |
| PDQ-12 | 0.044 | 0.000 | 0.000 | 0.000 | 0.000 | 0.000 | 0.069 | 0.004 | 0.000 | 0.000 | 0.000 | 0.000 | 0.104 | 0.036 | 0.001 | 0.030 | 0.181 | 0.135 | 0.087 | 0.089 | 0.000 |  |  |  |  |  |  |  |  |
| PDQ-13 | 0.071 | 0.033 | 0.032 | 0.067 | 0.000 | 0.000 | 0.000 | 0.000 | -0.009 | 0.049 | 0.047 | 0.055 | 0.011 | 0.042 | 0.010 | 0.000 | 0.047 | 0.178 | 0.044 | 0.008 | 0.117 | 0.000 |  |  |  |  |  |  |  |
| PDQ-14 | 0.000 | 0.000 | 0.000 | 0.000 | 0.000 | 0.000 | 0.000 | 0.008 | 0.007 | 0.025 | 0.009 | 0.006 | 0.097 | 0.000 | 0.000 | 0.110 | 0.009 | 0.006 | 0.220 | 0.000 | 0.048 | 0.056 | 0.000 |  |  |  |  |  |  |
| PDQ-15 | 0.000 | 0.000 | 0.000 | -0.010 | 0.000 | 0.000 | -0.022 | 0.011 | 0.000 | 0.023 | 0.030 | 0.072 | 0.000 | 0.000 | 0.000 | 0.079 | 0.000 | 0.000 | 0.020 | 0.062 | 0.018 | 0.000 | 0.140 | 0.000 |  |  |  |  |  |
| PDQ-16 | 0.000 | 0.034 | 0.000 | 0.000 | 0.032 | 0.005 | 0.000 | 0.023 | 0.040 | 0.000 | 0.000 | 0.000 | 0.000 | 0.106 | 0.000 | 0.000 | 0.000 | 0.033 | 0.028 | 0.000 | 0.091 | 0.088 | 0.095 | 0.214 | 0.000 |  |  |  |  |
| PDQ-17 | 0.000 | 0.000 | 0.000 | 0.000 | 0.000 | 0.000 | 0.000 | 0.000 | 0.000 | 0.000 | 0.078 | 0.046 | 0.022 | 0.131 | 0.037 | 0.000 | 0.000 | 0.091 | 0.000 | 0.000 | 0.000 | 0.000 | 0.042 | 0.174 | 0.086 | 0.000 |  |  |  |
| PDQ-18 | 0.000 | 0.000 | 0.000 | 0.000 | 0.000 | 0.000 | 0.000 | 0.000 | 0.000 | 0.009 | 0.055 | 0.083 | 0.000 | 0.000 | 0.099 | 0.000 | 0.000 | 0.000 | 0.213 | 0.086 | 0.000 | 0.013 | 0.095 | 0.000 | 0.000 | 0.196 | 0.000 |  |  |
| PDQ-19 | 0.000 | 0.000 | 0.000 | 0.000 | 0.019 | 0.019 | 0.000 | 0.059 | 0.000 | 0.000 | 0.011 | 0.051 | 0.000 | 0.000 | 0.070 | 0.108 | 0.000 | 0.000 | 0.000 | 0.054 | 0.000 | 0.000 | 0.003 | 0.137 | 0.042 | 0.064 | 0.074 | 0.000 |  |
| PDQ-20 | 0.000 | 0.022 | 0.000 | 0.012 | 0.000 | 0.009 | 0.000 | 0.000 | 0.000 | 0.000 | 0.000 | 0.027 | 0.104 | 0.023 | 0.000 | 0.000 | 0.092 | 0.073 | 0.000 | 0.026 | 0.047 | 0.159 | 0.000 | 0.014 | 0.083 | 0.120 | 0.086 | 0.147 | 0.000 |


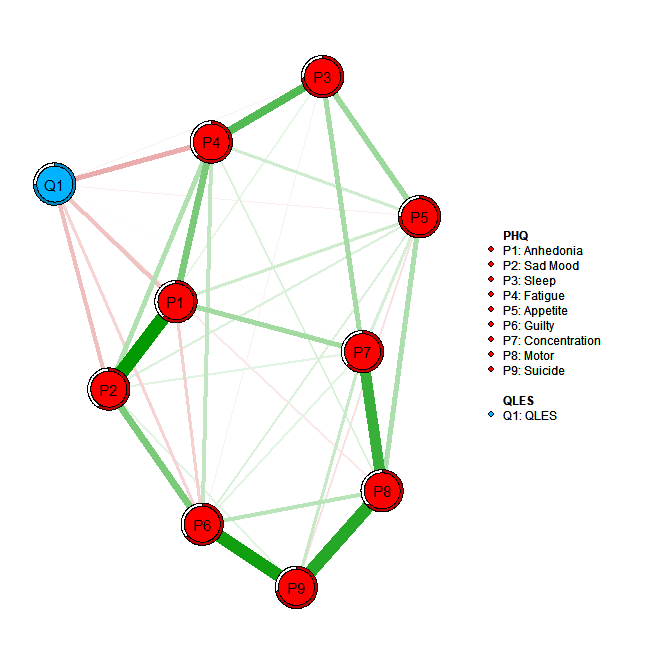
**Figure S1.** Estimated network model for the association between depressive symptoms and life satisfaction.


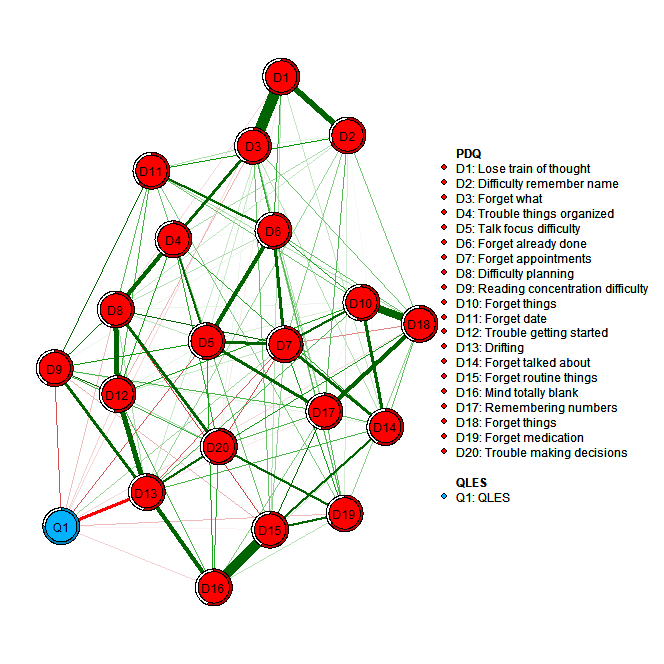
**Figure S2.** Estimated network model for the association between cognitive performance and life satisfaction.


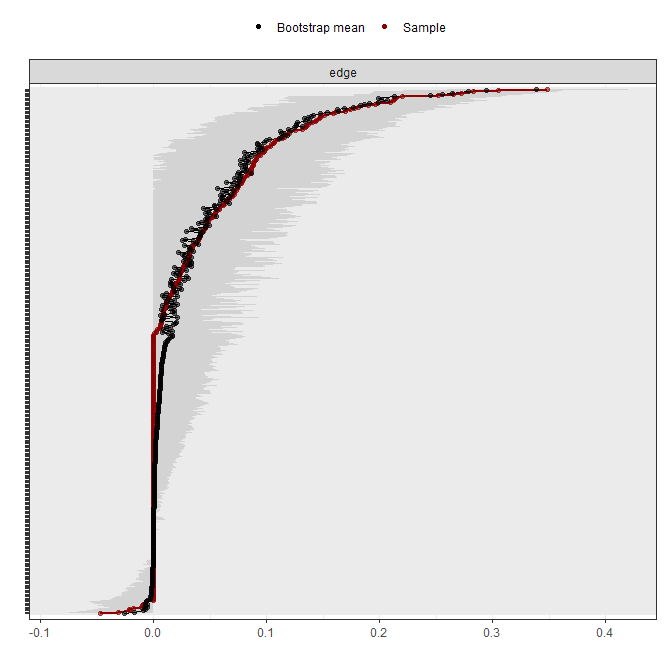


**Figure S3.** Nonparametric bootstrapped accuracy test for edge-weights of PHQ-9 and PDQ-D items

The black dots indicate the initial edge weights, whereas the red line corresponds to the bootstrapped values. The grey area corresponds to confidence intervals (CIs), with narrower grey area indicate more accurate edge weights.


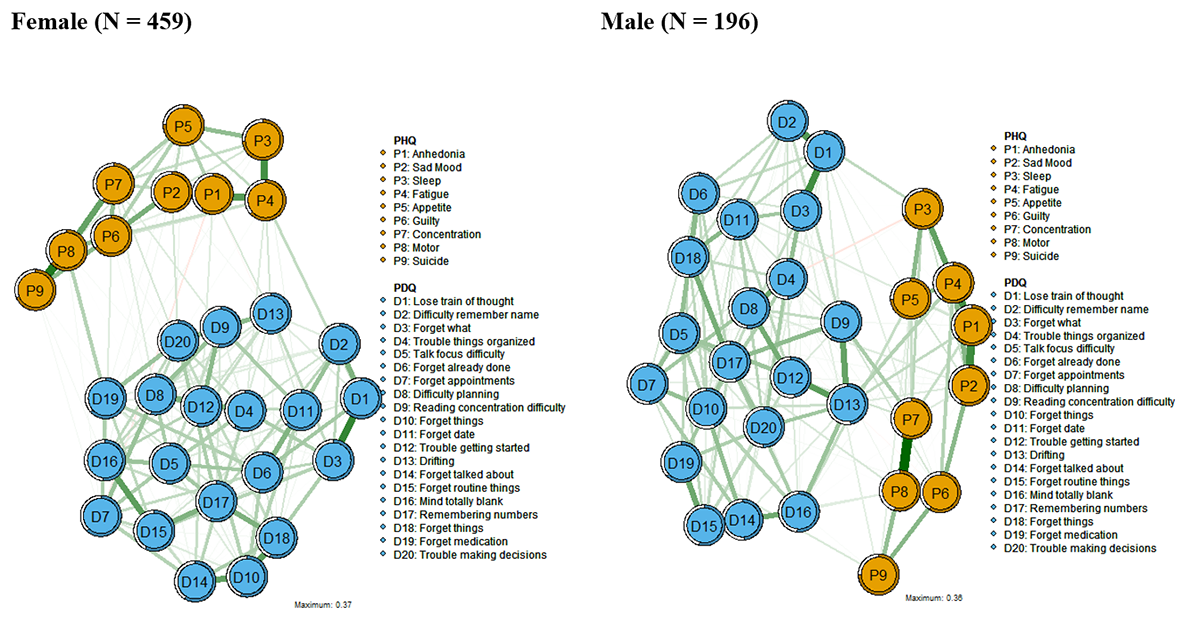
**Figure S4.** Estimated network models for depressive and cognitive impairment symptoms in male and female participants

The different-size circles represent different strength of the nodes, while the width and saturation of edges indicate the connections and directions (i.e., green: positive correlation; red: negative correlation). The ring around each node indicates the predictability (a fully filled dark ring would indicate that 100% of the symptom’s variance is explained by its intercorrelations with the other symptoms in the network). PDQ-D=Perceived Deficit Questionnaire for Depression, PHQ-9 = Patient Health Questionnaire.


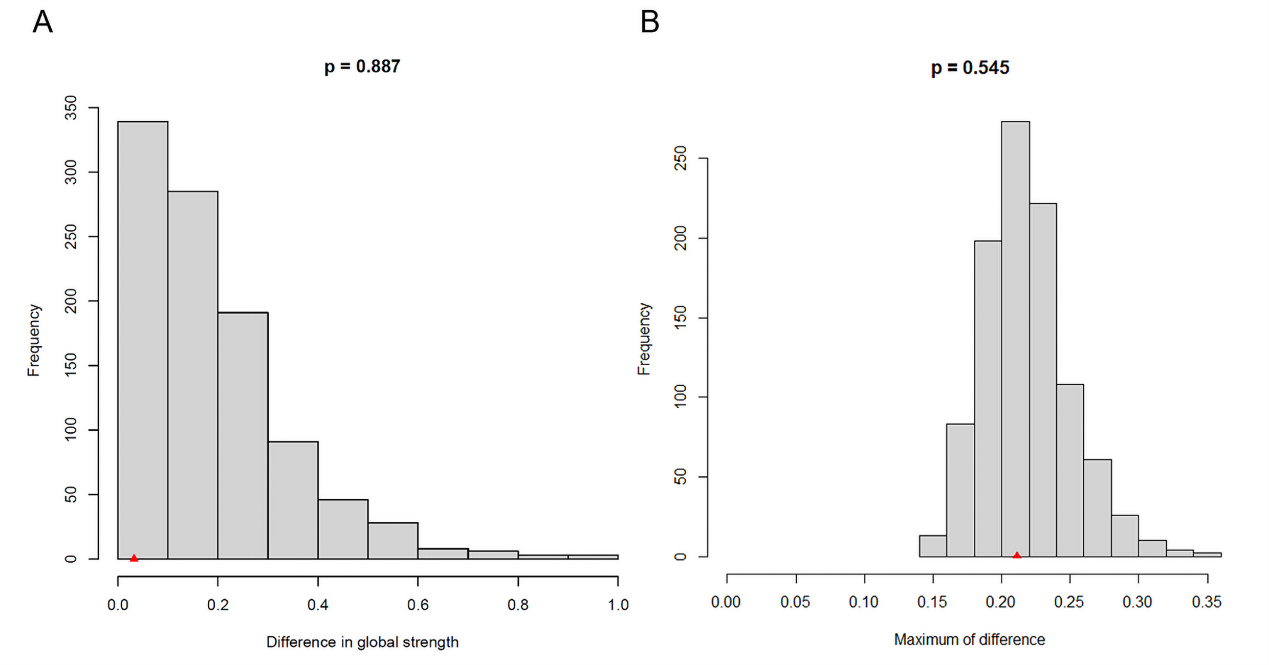


**Figure S5.** Statistical difference map of global strength and maximum of difference

The differences between the two networks (female vs male) were evaluated using the Network Comparison Test (NCT)

A．A plot of bootstrap value of the difference in network global strength. The difference was not significant (network strength among male participants: 13.83; among female participants: 13.87; S = 0.032, *p* = 0.887). Invariance in edges weights was examined using the permutation test, generating sets of p values for each edge-edge comparison. Holm-Bonferroni corrected p values were all > 0.05 indicating absence of significant differences.

B. A plot of bootstrap value of the maximum difference in any of the edge weights (1000 permutations). The difference was not significant (M = 0.21, *p* = 0.545).
